# Inducible Nitric Oxide Synthase Inhibition in the Medial Prefrontal Cortex Attenuates the Anxiogenic-Like Effect of Acute Restraint Stress *via* CB_1_ Receptors

**DOI:** 10.3389/fpsyt.2022.923177

**Published:** 2022-07-14

**Authors:** Arthur A. Coelho, Carla Vila-Verde, Ariandra G. Sartim, Daniela L. Uliana, Laura A. Braga, Francisco S. Guimarães, Sabrina F. Lisboa

**Affiliations:** ^1^Pharmacology Department, Ribeirão Preto Medical School-University of São Paulo, São Paulo, Brazil; ^2^Biomolecular Sciences Department, School of Pharmaceutical Sciences of Ribeirão Preto-University of São Paulo, São Paulo, Brazil; ^3^Departments of Neuroscience, Psychiatry and Psychology, University of Pittsburgh, Pittsburgh, PA, United States

**Keywords:** iNOS, CB_1_ receptor, medial prefrontal cortex, stress, anxiety

## Abstract

Stress exposure can result in several proinflammatory alterations in the brain, including overexpression of the inducible isoform of nitric oxide synthase (iNOS) in the medial prefrontal cortex (mPFC). These changes may be involved in the development of many psychiatric conditions. However, it is unknown if iNOS in mPFC plays a significant role in stress-induced behavioral changes. The endocannabinoid (ECB) system is also influenced by stress. Its activation seems to be a counter regulatory mechanism to prevent or decrease the stress-mediated neuroinflammatory consequences. However, it is unclear if the ECB system and iNOS interact to influence stress consequences. This study aimed to test the hypothesis that the anti-stress effect of iNOS inhibition in mPFC involves the local ECB system, particularly the CB_1_ cannabinoid receptors. Male Wistar rats with guide cannula aimed at the mPFC were submitted to acute restraint stress (RS) for 2 h. In the following morning, rats received bilateral microinjections of vehicle, AM251 (CB_1_ antagonist; 100 pmol), and/or 1400W (iNOS selective inhibitor; 10^−4^, 10^−3^, or 10^−2^ nmol) into the prelimbic area of mPFC (PL-mPFC) before being tested in the elevated plus-maze (EPM). iNOS inhibition by 1400W prevented the anxiogenic-like effect observed in animals submitted to RS. The drug did not promote behavior changes in naive animals, demonstrating a stress-dependent effect. The 1400W-anti-stress effect was prevented by local pretreatment with AM251. Our data suggest that iNOS inhibition may facilitate the local endocannabinoid signaling, attenuating stress effects.

## Introduction

Stress exposure is linked to a persistent stage of low inflammatory levels in the periphery and central nervous system, predisposing the individual to develop several pathologies like diabetes, cancer, and cardiovascular diseases (1–4). In this context, the disbalance of immunological components, combined with dysfunctional neuroendocrine and neurotransmitter systems, appears to contribute to psychiatric disorders, such as major depression, anxiety, schizophrenia, and posttraumatic stress disorder (PTSD) (4–11). Due to such relevance, many researchers worldwide turned their eyes to study the relationship between stress, neuroinflammation, and the development of psychiatric diseases.

Different stressors release danger/damage-associated molecular patterns (DAMPs) or “alarmins” involved in sterile inflammation, which can alter mood, increasing the risk of psychiatric disorders (12, 13). DAMPs, such as ATP, heat shock proteins, and high mobility group box1 (HMGB1) acting through pattern recognition receptors (PPRs), and proinflammatory mediators such as IL-1β, activate the transcription factor nuclear factor Kappa-B (NF-κB) (14). NF-κB is a significant regulator of the inducible nitric oxide synthase (iNOS) isoform (15). iNOS expression and activation increase nitric oxide (NO) synthesis (15), potentially resulting in oxidative/nitrosative stress and neurotoxicity (16).

NMDA receptors activation by glutamate release, which increases in the medial prefrontal cortex (mPFC) during and after stress (17–19), also activates NF-κB (20). Mice exposure to restraint stress (RS) associated with acoustic stress augments iNOS expression in the mPFC (21). Similarly, RS alone increased iNOS activity in the cortex of rats (20). Besides, genetic deletion of iNOS and its systemic pharmacological inhibition reduced stress-elicited behavioral consequences (22). Despite this evidence, the role of iNOS specifically in the mPFC in developing stress-related behaviors is not entirely understood. Previously we demonstrated that the inhibition of neuronal NOS (nNOS) in the prelimbic region of mPFC (PL-mPFC) reverted the anxiogenic-like effect induced by acute stress (23). However, the role of iNOS in this region in stress modulation is still poorly explored. Thus, our first aim is to test the hypothesis that, similar to nNOS, the local inhibition of iNOS in the PL-mPFC reduces the anxiogenic-like effect induced by acute stress.

Exposure to stressful events may also stimulate the endocannabinoid (ECB) system (24). This system can act through retrograde neuromodulators, the endocannabinoids (ECBs), which include anandamide (AEA) and 2-arachidonoylglycerol (2-AG). These two ECBs act on presynaptic CB_1_ receptors, reducing the Ca^2+^ influx and decreasing glutamate release (25, 26). They can also activate CB_2_ receptors, found predominantly in immune cells, like microglia (27), and show postsynaptic expression in neurons (28).

The ECB system is considered a stress-buffer system, preventing or attenuating stress-related emotional consequences. This neuromodulatory system also regulates proinflammatory mediators (29–32). Evidence suggests a functional interaction between the ECB and nitrergic systems in brain regions engaged by exposure to aversive stimuli (33–35), including in the mPFC (36). Moreover, CB_1_ KO mice have increased NOS activity in the brain (37) and pharmacological or genetic inhibition of cannabinoid receptors increases iNOS expression in the PFC after stress (31, 38). Therefore, iNOS inhibition may facilitate stress adaptation by favoring the ECB signaling. Thus, the second aim of this study is to test if the anti-stress effect of iNOS inhibition in the PL-mPFC depends on the CB_1_ signaling.

## Experimental Procedures

### Animals

**Figure 1:**
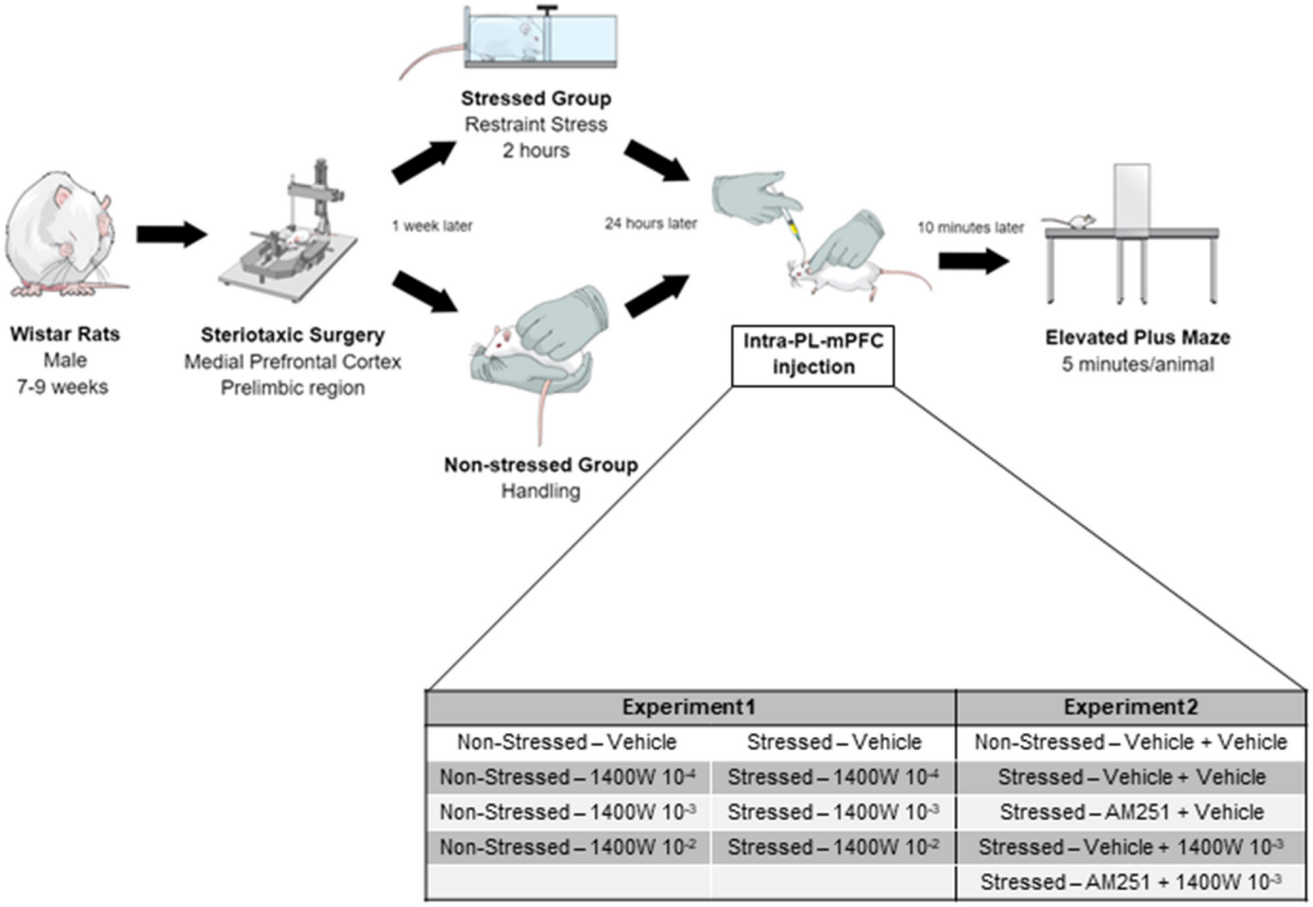
Timeline of all procedures performed. Stereotaxic surgery was performed to cannulae implantation into the PL-mPFC of all animals. After 1 week, the stressed groups were submitted to 2 h of restraint stress. The following morning, the animals received the intra-PL-mPFC injections and, after 10 min, were tested in the EPM. In experiment 2, the second injection was performed 5 min after the first one.

In the first part of this study (Experiment 1), male Wistar rats (240–300 g, 7–8 weeks old) were acquired from the animal facility of the University of São Paulo, campus Ribeirão Preto. However, this strain production was discontinued at Ribeirão Preto. Therefore, for Experiment 2 Wistar rats were obtained from the Anilab company (Paulinia, São Paulo, Brazil). Animals from different suppliers were not used in the same experiment. Rats were kept in the animal care unit of the Pharmacology Department (FMRP/USP). Rats were housed in groups of four animals per cage (acrylic boxes - 49 × 34 × 26 cm) in a temperature-controlled room (24 ± 1°C) under standard laboratory conditions: 12-h light/dark cycle (lights on at 06:00 a.m.), humidity of 50–55%, with free access to food (Nuvital, Nuvilab, Brazil) and filtered water. All experimental procedures, summarized in Figure 1, were approved by the Ethical Review Committee of the Medical School of Ribeirão Preto (protocol no. 224/2017). All efforts were made to reduce the suffering and the number of animals used in this study.

### Drugs

The selective, potent, and irreversible inhibitor of the iNOS enzyme, N-([3-(Aminomethyl)phenyl]methyl)ethanimidamide dihydrochloride (1400 W; Sigma-Aldrich, USA), at the doses of 10^−4^, 10^−3^, and 10^−2^ nmol/0.2 μL/side, was dissolved in sterile saline. 1400 W doses were calculated based on the Ki for nNOS of a selective nNOS inhibitor, n-propyl-l-arginine (NPLA; 0.01 nmol/0.2 μl;). The dose of 0.01 nmol/0.2 μl of NPLA in the mPFC attenuates immobility time in the forced swimming test (39). Based on this dose and the Ki of NPA (Ki = 57 nM) and 1400 W (Ki = 2,000 nM) for nNOS, we calculated the dose of 1400 W that would be equipotent to the dose of NPA to inhibit nNOS (~0.35 nmol). Considering that the Ki of 1400 W for inhibiting iNOS is around 286 times lower than that for inhibiting nNOS (7 nM), we calculated the dose of 0.0012 nmol (1.2 × 10^−3^ nmol) of 1400 W would selectively inhibit iNOS. Higher and lower 1400 W doses were also employed based on this last dose.

The CB_1_ receptor antagonist, N-(piperidin-1-yl)-5-(4-iodophenyl)-1-(2,4-dichlorophenyl)-4-methyl-1H-pyrazole-3-carboxamide (AM251, Tocris, USA), 100 pmol/0.2 μL/side, was dissolved in 10% dimethyl sulfoxide (DMSO) in saline (0.9% NaCl). This dose was based on previous data (40–43). Drugs were freshly prepared before use, kept on ice, and protected from light during experimental sessions. Each control group received the corresponding vehicle of the drug being tested (1400 W: saline, 0.2 μL/side, and AM251: 10% DMSO in saline, 0.2 μL/side).

For the stereotaxic surgery, we administered the general anesthetic 2,2,2-Tribromoethanol (2.5%, 1 ml/kg-Sigma-Aldrich, USA), the local anesthetic lidocaine (2%-Dentsply, Brazil), the poly antibiotic preparation of streptomycin and penicillin (1.200.000 UI−0.2 ml/rat; Pentabiotic-Fort Dodge, Brazil), and the anti-inflammatory flunixin meglumine (s.c., 0.025 g/kg; Banamine®-Schering Plough, Brazil) for postoperative analgesia.

### Stereotaxic Surgery

Five to seven days before the stress procedure, stereotaxic surgeries were performed in rodents to implant stainless steel guide cannula (11 mm, 0.6 mm outer diameter-OD) bilaterally into the pre-limbic region of the medial prefrontal cortex (PL-mPFC). Animals were previously anesthetized with 2,2,2-Tribromoethanol intraperitoneally (i.p.) and placed in the stereotaxic frame (Stoelting, USA). The skull was surgically exposed for cannulae implantation after scalp anesthesia with 2% lidocaine subcutaneous (s.c.). Based on the rat brain atlas (44), stereotaxic coordinates used were: AP: +3.3 mm from Bregma, L: +1.9 mm from the medial suture, V: −2.4 mm of the skull, and cannulae angle was 22°. The position of the incisor bar was adjusted to −3.2 mm. Guide cannulae were fixed to the skull with acrylic cement and a metal screw; a stylet inside the cannulae prevented obstruction. The animals received antibiotic (intramuscular – i.m. – Pentabiotic) and anti-inflammatory (s.c.-Banamine) injections during the surgery to avoid infections and induce analgesia.

### Restraint-Stress (RS)

One day before the behavioral test, animals were submitted to the restraint-stress (RS). Rodents were immobilized in metallic tubes (20 × 7 × 4 cm) with adjustable roof and ventilation holes for 2 h. The non-stressed animals received handling procedures during the restraint session. The protocol was conducted in a sound-attenuated and temperature-controlled (24°C) room. At the end of the RS, the animals (stressed and non-stressed) were individually housed in acrylic cages (36 × 25 × 24 cm) until the behavioral test. Handling and restraint stress were performed in the morning.

### Drug Microinjection

Animals received bilateral injections (0.2 μL each) into the PL-mPFC using a dental needle (33 G/0.3 mm OD), 1 mm longer than the guide cannula, connected via polyethylene tubing (PE-10) to a 10-μl syringe (7001-KH; Hamilton Co., USA). Needles were carefully inserted into the guide cannula, and the solution was infused for over 30 s with the help of an infusion pump (KD Scientific, Massachusetts, USA). The needles remained in the cannulae for an additional 30 s to prevent reflux.

### Elevated Plus Maze (EPM) and Experimental Design

All experiments were carried out between 7 a.m. and 2 p.m. Animals were transported to a sound-attenuated and temperature-controlled (24°C) room for habituation before starting experimental procedures. The injections were performed as described in the previous section. Ten minutes after the last injection, animals were subjected to the Elevated Plus Maze (EPM). The wood apparatus consists of two opposite open arms (50 cm length × 10 cm width), crossed at a right angle by two closed arms with the exact dimensions, enclosed by 40 cm high walls with no roof. The maze is 50 cm from the ground. The ANY-Maze^TM^ software (version 4.7, Stoelting) (RRID: SCR_014289) performed the behavior analysis of the movies recorded through a video camera. This software detects the animal's position within the maze by contrasting the animal's color with the maze floor and calculates the number of entries onto the arms and the time spent in the open arms. Each test lasted 5 min, and the apparatus was cleaned with an alcohol solution between trials.

All animals were habituated for at least 1 h in the experimental room. In the first experiment, rats received microinjections of vehicle (saline - 0.9% NaCl) or 1400 W (10^−4^, 10^−3^, or 10^−2^ nmol) into the PL-mPFC. 10 min later, they were tested on the EPM. Similarly, in the second experiment, animals received a first local injection of vehicle (10% DMSO in saline) or AM251 (100 pmol) followed, 5 min later, by the second injection of vehicle (saline-0.9% NaCl) or 1400 W (10^−3^ nmol) into the PL-mPFC. They were tested on the EPM 10 min later.

### Histological Procedure

**Figure 2:**
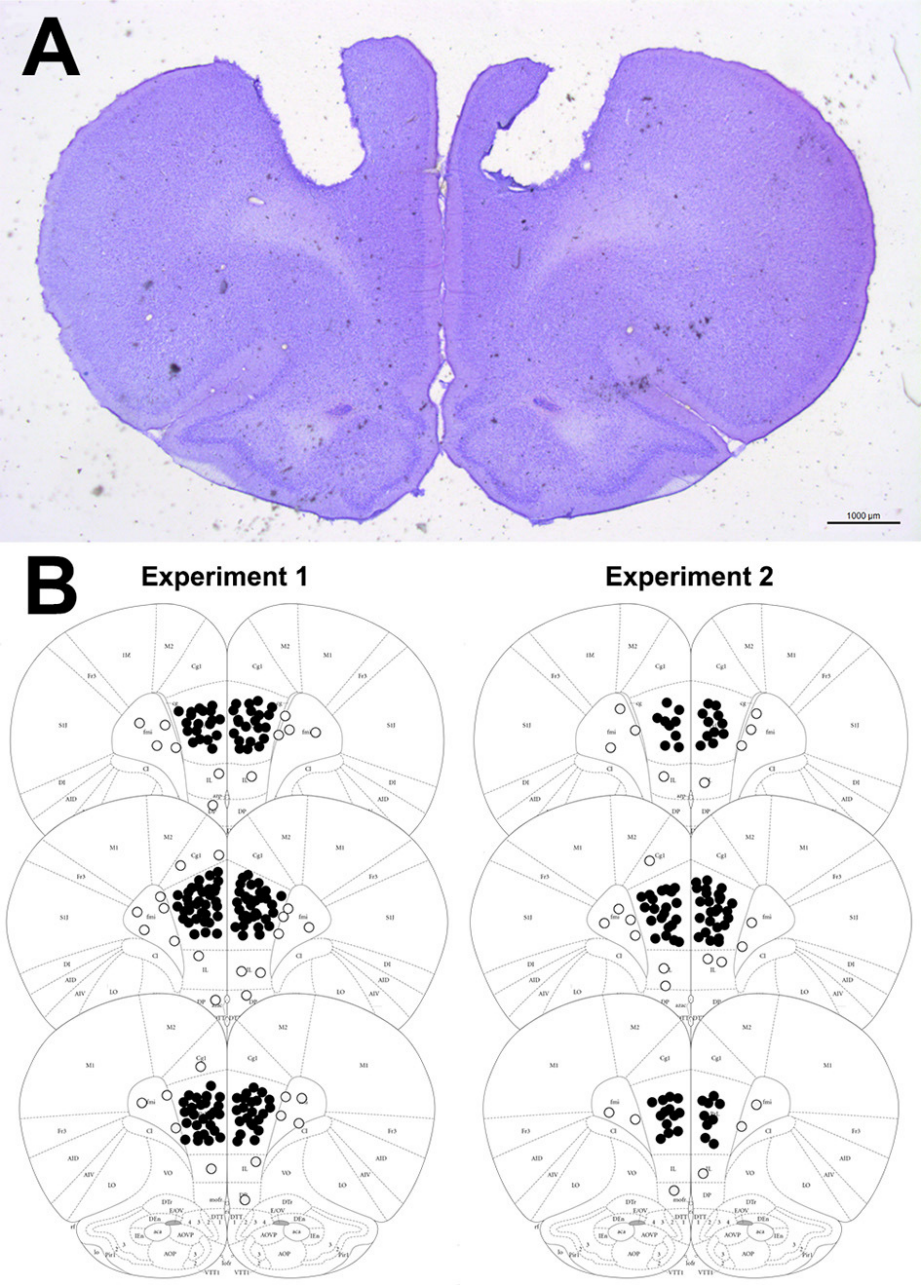
**(A)** Representative photomicrography showing bilateral microinjection site into the PL-mPFC (coronal slices). **(B)** Diagrammatic representation of coronal brain sections (based on Paxinos and Watson, 2006) with representative injection sites into the PL-mPFC (filled circles) and adjacent sites (empty circles). The number of circles represented in the picture is fewer than the actual number of animals used in the experiments since there is overlap.

In the afternoon that followed the behavioral tests, the rats were anesthetized with chloral hydrate 5% (1 mL/kg, i.p.) and perfused with isotonic saline followed by 10% formalin solution. As a site marker, 0.2 μL of 1% Evan's blue dye was bilaterally injected into the PL-mPFC. The brains were postfixed in 10% formalin solution for 24 h, and sections of 40 mm were cut using a cryostat (CM-1900; Leica, Wetzlar, Germany). The injection sites were identified with the help of the rat brain atlas (44). Representative photomicrography of the injection sites into the PL-mPFC can be seen in Figure 2. Rats receiving injections outside the aimed area were excluded from the analyses.

### Statistical Analysis

The anxiety index was calculated for each animal as in equation 1 (45):


$$\begin{array}{l} Anxiety\;index\\ =100-\left[\frac{\%\;entries\;in\;the\;open\;arms\;+\;\%\;time\;in\;the\;open\;arms}{2}\right] \end{array}$$

The percentage of entries and time in the open arms of the maze were plotted and analyzed separately.

All data were tested for normality and homogeneity of variance by the Kolmogorov-Smirnov and Levene tests, respectively. Welch's correction was performed to adjust the calculations of F and t ratios and degrees of freedom to adjust for heterogeneity of within-group variances. Behavioral parameters were expressed as the means ± standard error of the mean (SEM). For the first experiment, the anxiety index (percentage of entries and time in the open arm) and the number of enclosed arms entries were analyzed. First, all groups were analyzed by two-way Analysis of Variance (ANOVA), considering stress (condition) and treatment as factors to be analyzed. After that, naïve and stressed rats that received vehicle were compared by unpaired Student's *t*-test to verify stress interference. Then, each condition's data (naïve and stressed animals) were analyzed by one-way ANOVA followed by Tukey's *post hoc* test. For the second experiment, there was no drug administration in naïve rats. Therefore, naive and stressed-vehicle groups were compared by unpaired Student's *t*-test. After that, data from the stressed group were analyzed by one-way ANOVA followed by Tukey's *post hoc* test. Statistical differences were considered significant when *p* < 0.05. Statistical tendency was considered if *p* = 0.06–0.1.

## Results

### Intra-PL-mPFC Injection of 1400 W Reversed the Stress-Induced Anxiogenic-Like Effect in the Elevated Plus-Maze

**Figure 3:**
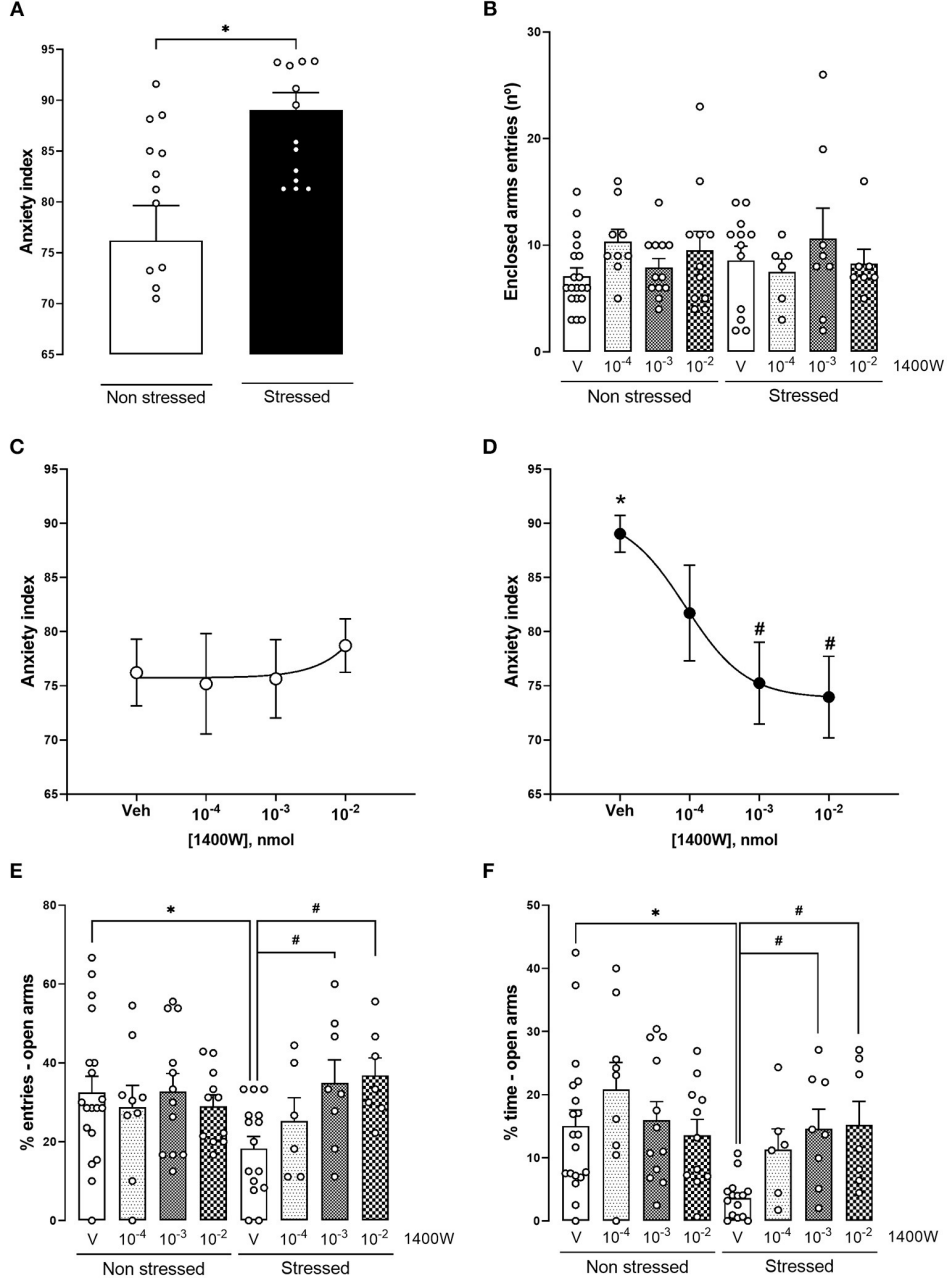
iNOS inhibition reversed the anxiety behavior induced by acute restraint stress (RS) 24 h later in the elevated plus-maze **(A)**. 1400 W microinjections did not affect naïve animals' behavior **(C)**. 1400 W microinjections, at the doses of 10^−3^ and 10^−2^ nmol in stressed animals, prevented the anxiogenic-like behavior represented by a decrease in the anxiety index **(D)** and increase in the percentage of entries **(E)** and time **(F)** in the open arms of the maze. Also, stress and the treatments did not alter the number of entries in the closed arms **(B)**. Each point/bar represents the mean ± standard error of the mean (SEM). *Indicates *p* <0.05 compared to the naïve vehicle group and ^#^Indicates *p* < 0.5 compared to the stressed vehicle group (one-way analysis of variance (ANOVA) followed by Tukey's *post hoc* test). *n* = 19, 9, 12, and 11 for naïve animals treated with vehicle, 1400 W 10^−4^, 1400 W 10^−3^, and 1400 W 10^−2^, respectively, and 15, 6, 8, and 7 for stressed animals treated with vehicle, 1400 W 10^−4^, 1400 W 10^−3^, and 1400 W 10^−2^, respectively.

In the anxiety index, a two-way ANOVA indicated a significant interaction between the factors (condition × treatment) [*F*_(3,79)_ = 2.866, *p* = 0.041, two-way ANOVA]. Comparing naïve-vehicle and stressed-vehicle, we observed that acute restraint stress (RS) induced an anxiogenic-like effect in the EPM 24 h later, increasing the anxiety index [*t*_(23.35)_ = 3.344, *p* = 0.002, unpaired *t*-test with Welch's correction] (Figure 3A). One-way ANOVA of stressed groups revealed that 1400 W (10^−3^ and 10^−2^ nmol) reversed the stress-induced anxiogenic-like effect [*F*_(3,32)_ =6.399, *p* = 0.001, one-way ANOVA;10^−3^ nmol: *p* = 0.007; 10^−2^ nmol: *p* = 0.004, Tukey's *post hoc*]. A dose-response curve was generated by non-linear regression (*r*^2^ = 0.374, *df* = 33) (Figure 3D). 1400 W did not alter behavior in non-stressed animals [*F*_(3,47)_ = 0.173, *p* = 0.914, one-way ANOVA] (Figure 3C). In addition, no changes were observed in the enclosed arm entries, suggesting that neither acute stress nor 1400 W affected basal motor activity (*p* > 0.05) (Figure 3B).

The analysis of the percentage of time and entries in the open arms of the EPM indicated the same conclusions. In the percentage of time spent in the open arms, there was an effect of condition [*F*_(1,79)_ = 5.820, *p* = 0.018] and treatment [*F*_(3,79)_ = 2.756, *p* < 0.049]. Moreover, there was a tendency for an interaction between the factors (condition × treatment) in the percentage of time [*F*_(3,79)_ = 2.450, *p* = 0.069] and entries [*F*_(3,79)_ = 2.545, *p* = 0.062] in the open arms. When comparing vehicle-treated groups, RS reduced the percentage of entries [*t*_(32)_ = 2.669, *p* = 0.011, unpaired *t*-test] and time [*t*_(21,55)_ = 4.253, *p* = 0.0003, unpaired *t*-test with Welch's correction] in the open arms, suggesting an anxiogenic-like effect. Doses of 10^−3^ and 10^−2^ reversed the reduction in the percentage of entries [*F*_(3,32)_ = 4.383, *p* = 0.010, one-way ANOVA] [10^−3^ nmol: *p* = 0.036; 10^−2^ nmol: *p* = 0.022, Tukey's *post hoc*] (Figure 3E), and percentage of time [*F*_(3,32)_ = 6.611, *p* = 0.001, one-way ANOVA] [10^−3^ nmol: *p* = 0.005; 10^−2^ nmol: *p* = 0.005, Tukey's *post hoc*] (Figure 3F) in the open arms of the EPM. None of the doses affected the non-stressed animals (*p* > 0.05).

### A CB_1_ Receptor Antagonist Injected Into the PL-mPFC Prevented 1400 W Effects on Stress-Induced Behavioral Consequences in the Elevated Plus-Maze

**Figure 4:**
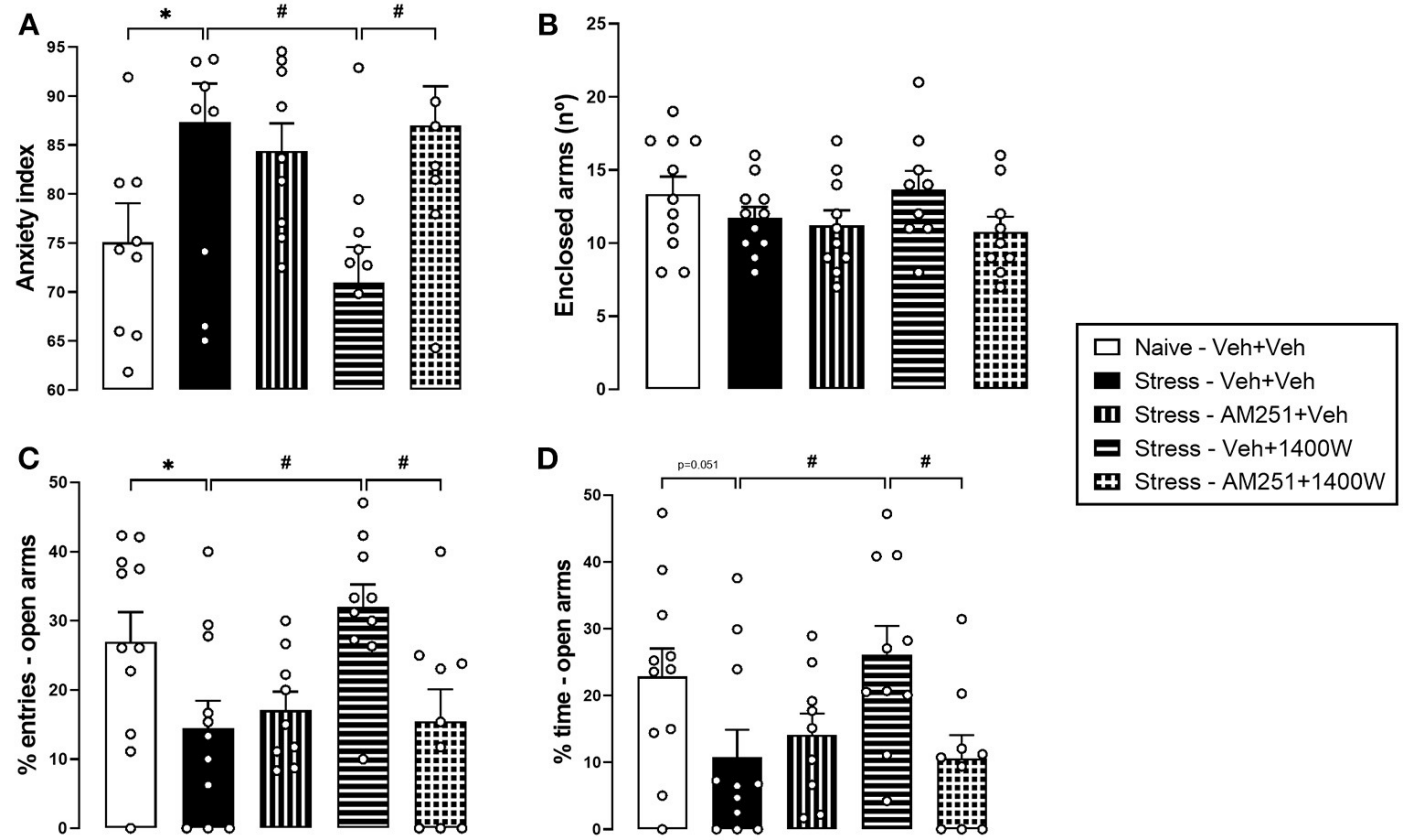
Cannabinoid type 1 (CB_1_) receptor antagonist prevented 1400 W effects on restraint stress-induced behavioral consequences in the elevated plus-maze (EPM) 24 h later. Pretreatment with the CB_1_ receptor antagonist AM251 (100 pmol) suppressed the 1400 W effect in the anxiety index **(A)** and percentage of entries **(C)** and time **(D)** in the open arms of the maze. There was no change in the enclosed arm entries **(B)**. Bars represent the mean ± standard error of the mean (SEM). *Indicates *p* < 0.05 in the Student *t*-test; ^#^Indicates *p* < 0.05 in Tukey *post-hoc* test. *n* = 11, 11, 9, 10, and 9 for Naïve-Veh+Veh, Stress-Veh+Veh, Stress-Am251+Veh, Stress-Veh+1400 W and Stress-AM251+1400 W, respectively.

Once again, RS induced an anxiogenic-like effect in the EPM, evidenced by the increase in the anxiety index [*t*_(20)_ = 2.206, *p* = 0.039]. One-way ANOVA also revealed a significant effect of treatment in stressed animals [*F*_(3,35)_ = 4.584, *p* = 0.008]. Confirming our previous result, the 1400 W injection (lower effective dose: 10^−3^ nmol) reversed the stress effect (*p* = 0.012, Tukey's *post hoc*). Prior intra-PL-mPFC injection of the CB_1_ receptor antagonist AM251 blocked the 1400 W effect (*p* > 0.999 compared with Stress–Veh+Veh group; and *p* = 0.021 compared with Stress–Veh+1400 W group, Tukey *post hoc*). AM251 did not induce any effect *per se* (*p* = 0.939, Tukey *post hoc*) (Figure 4A). No significant difference was observed in the enclosed arm entries (*p* > 0.05) (Figure 4B).

Similar conclusions were reached from the analysis of the percentage of entries and time in the open arms of the maze individually. RS increased the percentage of entries [*t*_(20)_ = 2.148, *p* = 0.044] and tended to increase the percentage of time [*t*_(20)_ = 2.075, *p* = 0.051] in the open arms of the EPM. The one-way ANOVA revealed a significant effect of treatment in stressed animals [%entries: *F*_(3,35)_ = 5.004, *p* = 0.005; %time: *F*_(3,35)_ = 3.686, *p* = 0.020]. 1400 W reversed the stress-induced anxiogenic-like effect (%entries: *p* = 0.008; %time: *p* = 0.030, Tukey's *post hoc*). The previous administration of AM251 blocked 1400 W effect (%entries: *p* = 0.997; %time: *p* > 0.999, Tukey's *post hoc*). AM251 had no effect *per se* (%entries: *p* = 0.957; %time: *p* = 0.930, Tukey's *post hoc*) (Figures 4C,D).

## Discussion

Our study confirms previous data showing that acute RS in rats induced anxiogenic-like behavior (23, 46–48). The administration of the selective inhibitor of iNOS 1400 W into the PL-mPFC reversed this anxiogenic effect but did not affect unstressed animals, suggesting that iNOS was activated by stress. Furthermore, we observed that the anti-stress effect of 1400 W is blocked by the antagonist AM251, indicating that it depends on the endocannabinoid signaling, particularly CB_1_ receptors.

RS induces several neurotransmitter changes. It increases glutamate levels (17, 48), alters the release of corticosterone (49, 50), and changes proinflammatory cytokines levels by activating NF-?B (51, 52). In addition, depending on the duration and the number of episodes, stress induces behavioral changes, including depressive- (51) and anxious-like (23, 46–48) behaviors. Our results showed that 2-h of RS decreased the exploration of open arms of EPM (percentage of entries and time), increasing the anxiety index. Also, stress did not alter the number of entries in the closed arms of EPM, suggesting that this was not a motor effect.

The PFC is connected to several brain areas and promotes the integration between stimuli, playing a pivotal role in decision making, goal-directed behaviors, and working memory (53, 54). However, the precise role of each of its subdivisions in these processes is still under debate. The PL-mPFC projects to the “stress circuit areas,” such as the basolateral amygdala (54, 55). It seems to be involved with stress response control and anxiety-related behaviors (55, 56). Stress exposure can promote plastic changes in the PL-mPFC (57, 58), increasing the expression of c-fos protein (59) or the firing rate of local neurons during acute stress (60), for example. Furthermore, stress increases monoamines, glutamate, and glucocorticoid release (17, 61, 62) and alters the dendritic morphology of PL (63). Finally, stressful events increase the expression and activity of iNOS in the mPFC (21, 64, 65). These findings suggest that PL-mPFC hyperactivation in response to stress could influence behavior and contribute to the anxiogenic effect. Moreover, as observed in the present work with iNOS, modulation of the local changes induced by stress could reverse the behavioral consequences of stress exposure. Previous studies (66, 67), including from our group (68), showed that systemic administration of iNOS inhibitors could induce anxiolytic-like effects, attenuate stress effects, or induce antidepressant-like effects. Altogether, the present results suggest that the mPFC could be a possible target of these drugs.

NO modulates several functions in the CNS, such as neurotransmitter release, synaptic plasticity, and neuroprotection (69–71). However, when at high concentrations, it can produce harmful actions (72). Increased NO levels, as observed in stressful situations, positively modulate glutamate release, which could create positive feedback and facilitate excitotoxicity (73, 74). Furthermore, NO can promote S-nitrosylation of cysteine thiol groups of several proteins, interfering with their functions (72, 75, 76). For example, the S-nitrosylation of protein-disulphide isomerase (PDI) in patients with neurodegenerative disorders inhibits its enzymatic activity, leading to the accumulation of polyubiquitinated proteins in the brain (77). Moreover, S-nitrosylation can result in protein misfolding, mitochondrial dysfunction, synaptic damage, and neuronal cell death (77). Overall, these changes induced by NO could negatively impact behavior.

Acute intense stress increased NO metabolites (NOx) levels in the PL-mPFC and induced anxiety-like behavior (78). Also, anxiety-like behavior caused by acute restraint stress was reverted by intra-PL-mPFC injection of an nNOS inhibitor (23). Moreover, intra-hippocampal injection of aminoguanidine, an iNOS inhibitor, prevented depressive behaviors induced by chronic stress (79). As far as we know, this is the first study demonstrating that, similarly to the latter study, inhibition of the iNOS enzyme directly in the PL-mPFC reverses the stress-induced anxiogenic-like effect. Considering previous evidence observed with nNOS inhibitors administered into the same brain region (23), the present data adds more complexity to NO's regulatory mechanisms in the mPFC, implicating iNOS involvement in the anxiogenic-like effect induced by stress. It is worth mentioning that the calculated dose of 1400W was based on its Ki values, which is almost 300 times lower to inhibit iNOS than for inhibiting nNOS. Therefore, considering 1400 W high selectivity for iNOS, it is unlikely that the doses used promote nNOS inhibition, strengthening the involvement of iNOS in the present study. The complexity of NO signaling could be attributed in part to the expression of NOS isoforms by different cell types, but also by several mechanisms by which NO can affect proteins and systems function (80). For future studies, it would be interesting to perform a combination of subeffective doses of the different isoform inhibitors to evaluate the possible attenuation of the stress effect.

The effect of 1400 W in the PL-mPFC was only observed in stressed rats, suggesting that even if iNOS is constitutively expressed in some brain areas in the healthy brain (81), stress exposure increases its expression or activity, which would be necessary to observe the behavioral changes. Therefore, the 1400 W effect is dependent on stress-induced molecular alterations.

The NO signaling can regulate and be regulated by the ECB system (33–35, 74, 80, 82). For example, it is well-established that CB_1_ receptor activation reduces glutamatergic neurotransmission, which could be important to avoid the previously mentioned excitotoxicity induced by NO and glutamate (74). Moreover, activation of CB receptors by anandamide in microglia attenuates iNOS activation via activation of the MAPK phosphatase-1 (MKP-1) (83). In addition, the overexpression of CB_1_ receptors in the spinal cord reduced the expression of NF-κB, TLR4, and IL-17 in this region (84). On the other hand, NO can regulate the activity of G protein-coupled receptors via S-nitrosylation of cysteine residues (75, 76). It was shown in a study with brain slices that after treatment with S-nitrosothiols, CB_1_ receptor agonists had reduced efficacy in ^35^S GTP Binding Assay (85). Therefore, we speculate that stress-induced iNOS activation and increase in NO levels could S-nitrosylate CB_1_ receptors, impairing its signaling in the mPFC. This possibility, however, still needs to be addressed.

The CB_1_ receptor is expressed in the mPFC (40, 86), and its activation usually attenuates anxiety-like behaviors in stressful conditions (38, 40). Moreover, a CB_1_ agonist decreased stress-induced iNOS expression in the cortex of mice (87). Finally, CB_1_ KO mice have increased NOS activity in the hippocampus (37). Together, these results suggest that the nitrergic and endocannabinoid systems could play opposite roles in regulating stress-induced anxiety behavior in the PL-mPFC. Corroborating this proposal, we found that the anti-stress effect of 1400 W in this region was blocked by the previous administration of the CB_1_ antagonist AM251.

iNOS is constitutively expressed in neurons in some brain regions (81) and astrocytes in cortical tissue (88). However, its expression increases significantly in microglia after inflammatory stimuli (81). Psychological stressors activate microglia (89) and increase iNOS expression in the brain, particularly in the PFC (64). Increased NO production is involved in stress response (23, 29, 78). Therefore, it is possible that the anti-stress effect of 1400 W results from inhibition of iNOS activity and NO production in microglial cells, reducing the neuronal impact of stress. However, we cannot exclude that iNOS inhibition also occurs in neurons.

Microglia cells synthesize and release ECBs (90, 91), and stress activates the ECB system (98). Then, iNOS inhibition may facilitate the ECB signaling, buffering the stress response. Accordingly, AM251 reversed the anti-stress effect of 1400 W. Considering that CB_1_ receptors are predominantly expressed by neurons in the brain and that a CB_1_ antagonist reversed the 1400 W effect, we suggest that, in the presence of an iNOS inhibition, local ECBs could regulate neuronal excitability through CB_1_ receptors and prevent the manifestation of the stress response in the EPM.

**Figure 5:**
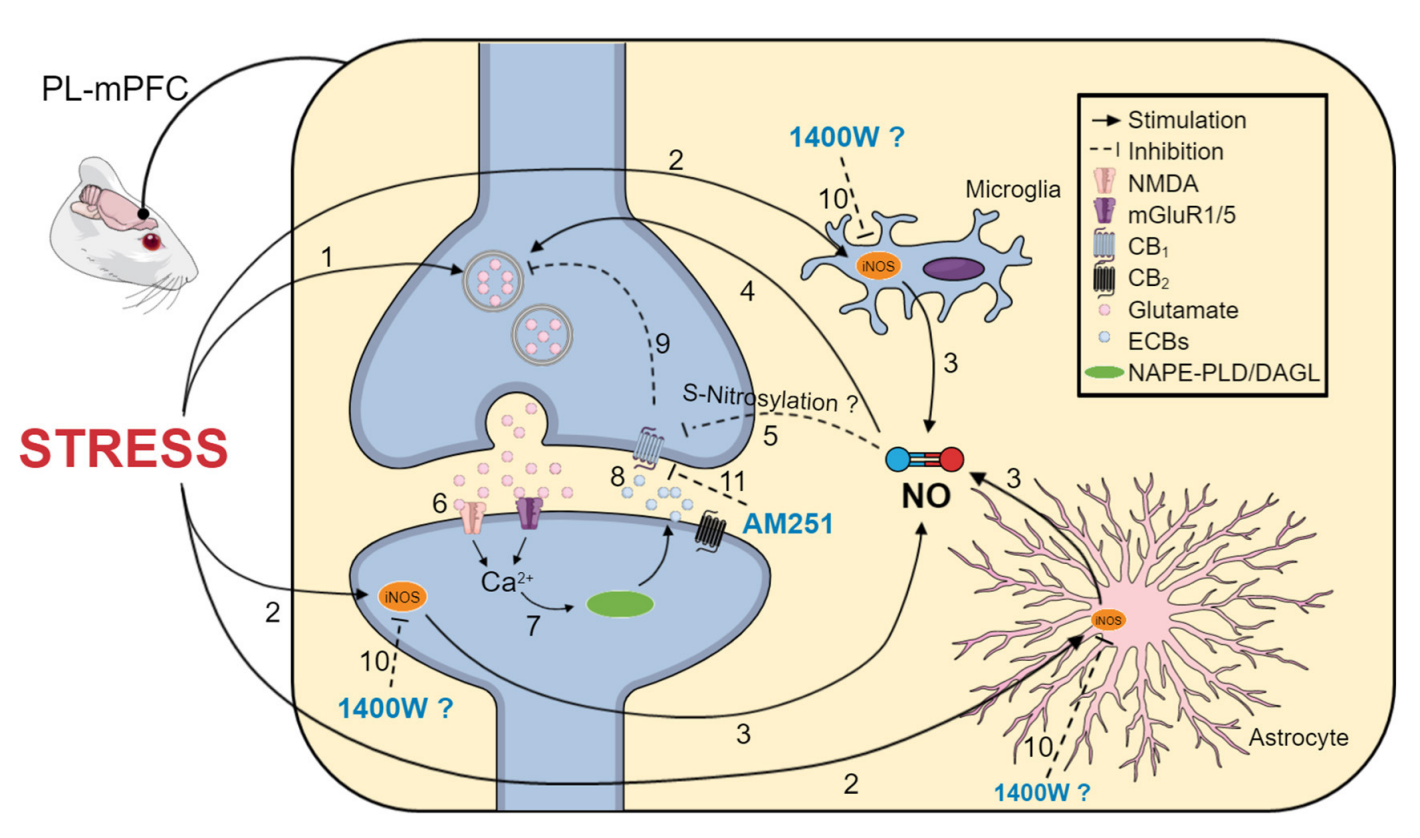
Schematic representation of the proposed mechanisms involved in the anti-stress effect of 1400 W in the PL-mPFC. (1) Acute stress induces glutamate release (2) and activates iNOS, which could be expressed in microglia, astrocytes, and neurons. (3) Increased iNOS activity increases NO levels, (4) which can potentiate glutamatergic transmission. (5) NO may promote protein S-nitrosylation, including the CB_1_ receptor. Therefore, it could inhibit its function, impairing neurotransmission control. (6) Glutamate also activates mGlu5 and NMDA receptors, increasing intracellular calcium levels, (7) resulting in activation of the ECB synthesizing enzymes, NAPE-PLD and DAGL-a, increasing ECB levels. (8) Anandamide and 2-AG could act on CB_1/2_ receptors (9), regulating neuronal excitability. In the context of stress, NO could overcome ECBs' stress-buffer actions, favoring the release of glutamate, excessive excitability, and anxiety-like behaviors. In this way, (10) iNOS inhibition by 1400W could attenuate this positive feedback in neurons and reduce inflammatory consequences of iNOS activation in glial cells. ECBs acting at CB_1_ receptors contributes to the anxiolytic effect of 1400 W because (11) blocking CB_1_ receptors with AM251 prevented the anti-stress effect of 1400 W. We propose that the anti-stress effect of pharmacological iNOS inhibition in the PL-mPFC is dependent on the local ECB signaling, mediated at least by the CB_1_ receptors. The figure was simplified. Therefore, not all cells and components of synapses and glial cells are depicted.

In conclusion, our results indicate that the iNOS in the PL-mPFC is also involved in the behavioral consequences of acute stress exposure. Moreover, the present data strengthens the proposal that there is a crosstalk between the nitrergic and endocannabinoid systems, particularly CB_1_ receptors, in modulating stress and anxiety behaviors. As summarized in Figure 5, we propose that the behavioral effects after restraint stress may be caused by increased glutamate release, which is potentiated by NO synthesis from iNOS. NO could promote S-nitrosylation of several proteins, including CB_1_. In this case, it could inhibit CB_1_ function, impairing neurotransmission control. Under homeostatic conditions, the activation of the endocannabinoid system, resulting in the release of anandamide and 2-AG, could be able to counteract the stress effects. However, when this signaling is impaired, the anxiogenic effect prevails. By inhibiting iNOS, we can attenuate the positive feedback mentioned before, allowing the endocannabinoid activity to regulate the synaptic neurotransmission, resulting in an anxiolytic effect.

## Nomenclature

Resource Identification Initiative ANY-maze (RRID: SCR_014289).

## Data Availability Statement

The raw data supporting the conclusions of this article will be made available by the authors, without undue reservation.

## Ethics Statement

The animal study was reviewed and approved by Ethical Review Committee, Ribeirão Preto Medical School (protocol no. 224/2017).

## Author Contributions

SL and FG designed the study. AC, CV-V, AS, DU, and LB performed the experiments and developed the data analysis. AC and CV-V wrote the manuscript. All authors approved the final version.

## Funding

This work was supported by the São Paulo Research Foundation (FAPESP, Grant numbers: 2017/24304-0 and 2017/19731-6), CAPES (PROEX) and CNPq.

## Conflict of Interest

The authors declare that the research was conducted in the absence of any commercial or financial relationships that could be construed as a potential conflict of interest.

## Publisher's Note

All claims expressed in this article are solely those of the authors and do not necessarily represent those of their affiliated organizations, or those of the publisher, the editors and the reviewers. Any product that may be evaluated in this article, or claim that may be made by its manufacturer, is not guaranteed or endorsed by the publisher.

## References

[1] Farah C Michel LYM Balligand JL. Nitric oxide signalling in cardiovascular health and disease. Nat Rev Cardiol. (2018) 15:292–316. 10.1038/nrcardio.2017.224 29388567

[2] Ptaschinski C Lukacs NW. Acute and chronic inflammation induces disease pathogenesis., in Molecular Pathology: The Molecular Basis of Human Disease. Elsevier Inc. (2018). p. 25–43 10.1016/B978-0-12-802761-5.00002-X

[3] Haroon E Raison CL Miller AH. Psychoneuroimmunology meets neuropsychopharmacology: translational implications of the impact of inflammation on behavior. Neuropsychopharmacology. (2012) 37:137–62. 10.1038/npp.2011.205 21918508 PMC3238082

[4] Miller AH Haroon E Felger JC. Therapeutic implications of brain-immune interactions: treatment in translation. Neuropsychopharmacology. (2017) 42:334–59. 10.1038/npp.2016.167 27555382 PMC5143492

[5] Jones KA Thomsen C. The role of the innate immune system in psychiatric disorders. Mol Cell Neurosci. (2013) 53:52–62. 10.1016/j.mcn.2012.10.002 23064447

[6] Beumer W Gibney SM Drexhage RC Pont-Lezica L Doorduin J Klein HC. The immune theory of psychiatric diseases: a key role for activated microglia and circulating monocytes. J Leukoc Biol. (2012) 92:959–75. 10.1189/jlb.0212100 22875882

[7] Leonard BE. Inflammation, depression and dementia: are they connected? Neurochem Res. (2007) 32:1749–56. 10.1007/s11064-007-9385-y 17705097

[8] Leonard BE. Impact of inflammation on neurotransmitter changes in major depression: an insight into the action of antidepressants. Prog Neuropsychopharmacol Biol Psychiatry. (2014) 48:261–7. 10.1016/j.pnpbp.2013.10.018 24189118

[9] Leonard BE Schwarz M Myint AM. The metabolic syndrome in schizophrenia: is inflammation a contributing cause? J Psychopharmacol. (2012) 26:33–41. 10.1177/0269881111431622 22472311

[10] Na KS Jung HY Kim YK. The role of pro-inflammatory cytokines in the neuroinflammation and neurogenesis of schizophrenia. Prog Neuropsychopharmacol Biol Psychiatry. (2014) 48:277–86. 10.1016/j.pnpbp.2012.10.022 23123365

[11] Rohleder N Joksimovic L Wolf JM Kirschbaum C. Hypocortisolism and increased glucocorticoid sensitivity of pro-Inflammatory cytokine production in Bosnian war refugees with posttraumatic stress disorder. Biol Psychiatry. (2004) 55:745–51. 10.1016/j.biopsych.2003.11.018 15039004

[12] Franklin TC Xu C Duman RS. Depression and sterile inflammation: essential role of danger associated molecular patterns. Brain Behav Immun. (2018) 72:2–13. 10.1016/j.bbi.2017.10.025 29102801

[13] Troubat R Barone P Leman S Desmidt T Cressant A Atanasova B. Neuroinflammation and depression: a review. Eur J Neurosci. (2021) 53:151–71. 10.1111/ejn.14720 32150310

[14] Gadani SP Walsh JT Lukens JR Kipnis J. Dealing with danger in the CNS: the response of the immune system to injury. Neuron. (2015) 87:47–62. 10.1016/j.neuron.2015.05.019 26139369 PMC4491143

[15] Aktan F. iNOS-mediated nitric oxide production and its regulation. Life Sci. (2004) 75:639–53. 10.1016/j.lfs.2003.10.042 15172174

[16] Zlatković J Filipović D. Bax and B-cell-lymphoma 2 mediate proapoptotic signaling following chronic isolation stress in rat brain. Neuroscience. (2012) 223:238–45. 10.1016/j.neuroscience.2012.08.005 22885231

[17] Moghaddam B. Stress preferentially increases extraneuronal levels of excitatory amino acids in the prefrontal cortex: comparison to hippocampus and basal ganglia. J Neurochem. (1993) 60:1650–7. 10.1111/j.1471-4159.1993.tb13387.x 8097232

[18] Moghaddam B Bolinao ML Stein-Behrens B Sapolsky R. Glucocortcoids mediate the stress-induced extracellular accumulation of glutamate. Brain Res. (1994) 655:251–4. 10.1016/0006-8993(94)91622-5 7812782

[19] Bagley J Moghaddam B. Temporal dynamics of glutamate efflux in the prefrontal cortex and in the hippocampus following repeated stress: effects of pretreatment with saline or diazepam: *Neuroscience*. (1997) 77:65–73. 10.1016/S0306-4522(96)00435-6 9044375

[20] Madrigal JLM Moro MA Lizasoain I Lorenzo P Castrillo A Boscá L. Inducible nitric oxide synthase expression in brain cortex after acute restraint stress is regulated by nuclear factor κB-mediated mechanisms. J Neurochem. (2001) 76:532–8. 10.1046/j.1471-4159.2001.00108.x 11208916

[21] Gárate I Garcia-Bueno B Madrigal JLM Caso JR Alou L Gomez-Lus ML. Stress-induced neuroinflammation: role of the toll-like receptor-4 pathway. Biol Psychiatry. (2013) 73:32–43. 10.1016/j.biopsych.2012.07.005 22906518

[22] Montezuma K Biojone C Lisboa SF Cunha FQ Guimarães FS Joca SRL. Inhibition of iNOS induces antidepressant-like effects in mice: pharmacological and genetic evidence. Neuropharmacology. (2012) 62:485–91. 10.1016/j.neuropharm.2011.09.004 21939674

[23] Vila-Verde C Marinho ALZ Lisboa SF Guimarães FS. Nitric oxide in the prelimbic medial prefrontal cortex is involved in the anxiogenic-like effect induced by acute restraint stress in rats. Neuroscience. (2016) 320:30–42. 10.1016/j.neuroscience.2016.01.040 26812037

[24] Riebe CJ Wotjak CT. Endocannabinoids and stress. Stress. (2011) 14:384–97. 10.3109/10253890.2011.586753 21663537

[25] Cristino L Bisogno T Di Marzo V. Cannabinoids and the expanded endocannabinoid system in neurological disorders. Nat Rev Neurol. (2020) 16:9–29. 10.1038/s41582-019-0284-z 31831863

[26] Hillard CJ. Stress regulates endocannabinoid-CB1 receptor signaling. Semin Immunol. (2014) 26:380–8. 10.1016/j.smim.2014.04.001 24882055 PMC4247817

[27] Komorowska-Müller JA Schmöle AC. CB2 receptor in microglia: the guardian of self-control. Int J Mol Sci. (2021) 22:19. 10.3390/ijms22010019 33375006 PMC7792761

[28] Yin AQ Wang F Zhang X. Integrating endocannabinoid signaling in the regulation of anxiety and depression. Acta Pharmacol Sin. (2019) 40:336–41. 10.1038/s41401-018-0051-5 30002489 PMC6460364

[29] Lisboa SF Gomes FV Guimaraes FS Campos AC. Microglial cells as a link between cannabinoids and the immune hypothesis of psychiatric disorders. Front Neurol. (2016) 7:5. 10.3389/fneur.2016.00005 26858686 PMC4729885

[30] Lisboa SF Gomes FV Silva AL Uliana DL Camargo LHA Guimars FS. Increased contextual fear conditioning in inos knockout mice: additional evidence for the involvement of nitric oxide in stress-related disorders and contribution of the endocannabinoid system. Int J Neuropsychopharmacol. (2015) 18:pyv005. 10.1093/ijnp/pyv005 25618404 PMC4571624

[31] Zoppi S Madrigal JL Caso JR García-Gutiérrez MS Manzanares J Leza JC. Regulatory role of the cannabinoid CB2 receptor in stress-induced neuroinflammation in mice. Br J Pharmacol. (2014) 171:2814–26. 10.1111/bph.12607 24467609 PMC4243857

[32] Lisboa SF Niraula A Resstel LB Guimaraes FS Godbout JP Sheridan JF. Repeated social defeat-induced neuroinflammation, anxiety-like behavior and resistance to fear extinction were attenuated by the cannabinoid receptor agonist WIN55,212-2. Neuropsychopharmacology. (2018) 43:1924–33. 10.1038/s41386-018-0064-2 29786066 PMC6046035

[33] Lisboa SF Guimarães FS. Differential role of CB1 and TRPV1 receptors on anandamide modulation of defensive responses induced by nitric oxide in the dorsolateral periaqueductal gray. Neuropharmacology. (2012) 62:2455–62. 10.1016/j.neuropharm.2012.02.008 22394688

[34] Lisboa SF Magesto AC Aguiar JC Resstel LBM Guimarães FS. Complex interaction between anandamide and the nitrergic system in the dorsolateral periaqueductal gray to modulate anxiety-like behavior in rats. Neuropharmacology. (2013) 75:86–94. 10.1016/j.neuropharm.2013.07.008 23899460

[35] Lisboa SF Camargo LHA Magesto AC Resstel LBM Guimarães FS. Cannabinoid modulation of predator fear: involvement of the dorsolateral periaqueductal gray. Int J Neuropsychopharmacol. (2014) 17:1193–206. 10.1017/S1461145713001788 24438603

[36] Uliana DL Antero LS Borges-Assis AB Rosa J Vila-Verde C Lisboa SF. Differential modulation of the contextual conditioned emotional response by CB1 and TRPV1 receptors in the ventromedial prefrontal cortex: possible involvement of NMDA/nitric oxide-related mechanisms. J Psychopharmacol. (2020) 34:1043–55. 10.1177/0269881120928201 32638638

[37] Sun HK Seok JW Xiao OM Ledent C Jin K Greenberg DA. Role for neuronal nitric-oxide synthase in cannabinoid-induced neurogenesis. J Pharmacol Exp Ther. (2006) 319:150–4. 10.1124/jpet.106.107698 16831955

[38] Zoppi S Pérez Nievas BG Madrigal JLM Manzanares J Leza JC García-Bueno B. Regulatory role of cannabinoid receptor 1 in stress-induced excitotoxicity and neuroinflammation. Neuropsychopharmacology. (2011) 36:805–18. 10.1038/npp.2010.214 21150911 PMC3055736

[39] Pereira VS Romano A Wegener G Joca SRL. Antidepressant-like effects induced by NMDA receptor blockade and NO synthesis inhibition in the ventral medial prefrontal cortex of rats exposed to the forced swim test. Psychopharmacology. (2015) 232:2263–73. 10.1007/s00213-014-3853-2 25589143

[40] Lisboa SF Reis DG da Silva AL Corrěa FMA Guimarães FS Resstel LBM. Cannabinoid CB1 receptors in the medial prefrontal cortex modulate the expression of contextual fear conditioning. Int J Neuropsychopharmacol. (2010) 13:1163–73. 10.1017/S1461145710000684 20587131

[41] Lisboa SF Borges AA Nejo P Fassini A Guimarães FS Resstel LB. Cannabinoid CB1 receptors in the dorsal hippocampus and prelimbic medial prefrontal cortex modulate anxiety-like behavior in rats: additional evidence. Prog Neuropsychopharmacol Biol Psychiatry. (2015) 59:76–83. 10.1016/j.pnpbp.2015.01.005 25595265

[42] Lisboa SF Resstel LBM Aguiar DC Guimarães FS. Activation of cannabinoid CB1 receptors in the dorsolateral periaqueductal gray induces anxiolytic effects in rats submitted to the Vogel conflict test. Eur J Pharmacol. (2008) 593:73–8. 10.1016/j.ejphar.2008.07.032 18691568

[43] Resstel LBM Lisboa SF Aguiar DC Corrêa FMA Guimarães FS. Activation of CB1 cannabinoid receptors in the dorsolateral periaqueductal gray reduces the expression of contextual fear conditioning in rats. Psychopharmacology. (2008) 198:405–11. 10.1007/s00213-008-1156-1 18446325

[44] Paxinos G Watson C. The Rat Brain in Stereotaxic Coordinates. 6th ed. San Diego, CA: Academic Press (2006).

[45] Mazor A Matar MA Kaplan Z Kozlovsky N Zohar J Cohen H. Gender-related qualitative differences in baseline and post-stress anxiety responses are not reflected in the incidence of criterion-based PTSD-like behaviour patterns. World J Biol Psychiatry. (2009) 10:856–69. 10.1080/15622970701561383 17886167

[46] Resstel LBM Tavares RF Lisboa SFS Joca SRL Corrêa FMA Guimarães FS. 5-HT 1A receptors are involved in the cannabidiol-induced attenuation of behavioural and cardiovascular responses to acute restraint stress in rats. Br J Pharmacol. (2009) 156:181–8. 10.1111/j.1476-5381.2008.00046.x 19133999 PMC2697769

[47] Padovan CM del Bel EA Guimarães FS. Behavioral effects in the elevated plus maze of an NMDA antagonist injected into the dorsal hippocampus: influence of restraint stress. Pharmacol Biochem Behav. (2000) 67:325–30. 10.1016/S0091-3057(00)00361-0 11124397

[48] Orellana JA Moraga-Amaro R Díaz-Galarce R Rojas S Maturana CJ Stehberg J. Restraint stress increases hemichannel activity in hippocampal glial cells and neurons. Front Cell Neurosci. (2015) 9:102. 10.3389/fncel.2015.00102 25883550 PMC4382970

[49] Busnardo C Tavares RF Resstel LBM Elias LLK Correa FMA. Paraventricular nucleus modulates autonomic and neuroendocrine responses to acute restraint stress in rats. Auton Neurosci. (2010) 158:51–7. 10.1016/j.autneu.2010.06.003 20594922

[50] Busnardo C Alves FHF Crestani CC Scopinho AA Resstel LBM Correa FMA. Paraventricular nucleus of the hypothalamus glutamate neurotransmission modulates autonomic, neuroendocrine and behavioral responses to acute restraint stress in rats. Eur Neuropsychopharmacol. (2013) 23:1611–22. 10.1016/j.euroneuro.2012.11.002 23201369

[51] Salehpour F Farajdokht F Cassano P Sadigh-Eteghad S Erfani M Hamblin MR. Near-infrared photobiomodulation combined with coenzyme Q10 for depression in a mouse model of restraint stress: reduction in oxidative stress, neuroinflammation, and apoptosis. Brain Res Bull. (2019) 144:213–22. 10.1016/j.brainresbull.2018.10.010 30385146 PMC6309497

[52] Calcia MA Bonsall DR Bloomfield PS Selvaraj S Barichello T Howes OD. Stress and neuroinflammation: a systematic review of the effects of stress on microglia and the implications for mental illness. Psychopharmacology. (2016) 233:1637–50. 10.1007/s00213-016-4218-9 26847047 PMC4828495

[53] Kolb B. Functions of the frontal cortex of the rat: a comparative review. Brain Res. (1984) 320:65–98. 10.1016/0165-0173(84)90018-3 6440660

[54] Vertes RP. differential projections of the infralimbic and prelimbic cortex in the rat. Synapse. (2004) 51:32–58. 10.1002/syn.10279 14579424

[55] Jacobs DS Moghaddam B. Medial prefrontal cortex encoding of stress and anxiety. Int Rev Neurobiol. (2021) 158:29–55. 10.1016/bs.irn.2020.11.014 33785149

[56] Maaswinkel H Gispen W-H Spruijt BM. Effects of an electrolytic lesion of the prelimbic area on anxiety-related and cognitive tasks in the rat. Behav Brain Res. (1996) 79:51–9. 10.1016/0166-4328(95)00261-8 8883816

[57] Holmes A Wellman CL. Stress-induced prefrontal reorganization and executive dysfunction in rodents. Neurosci Biobehav Rev. (2009) 33:773–83. 10.1016/j.neubiorev.2008.11.005 19111570 PMC2941982

[58] Arnsten AFT. Stress weakens prefrontal networks: molecular insults to higher cognition. Nat Neurosci. (2015) 18:1376–85. 10.1038/nn.4087 26404712 PMC4816215

[59] Cullinan WE Herman JP Battaglia DF Akilt H Watsont SJ. Pattern and time course of immediate early gene expression in rat brain following acute stress. Neuroscience. (1995) 64:477–505. 10.1016/0306-4522(94)00355-9 7700534

[60] del Arco A Park J Moghaddam B. Unanticipated stressful and rewarding experiences engage the same prefrontal cortex and ventral tegmental area neuronal populations. eNeuro. (2020) 7:ENEURO.0029-20.2020. 10.1523/ENEURO.0029-20.2020 32385042 PMC7294461

[61] Ostrander MM Richtand NM Herman JP. Stress and amphetamine induce Fos expression in medial prefrontal cortex neurons containing glucocorticoid receptors. Brain Res. (2003) 990:209–14. 10.1016/j.brainres.2003.07.001 14568346

[62] Morrow BA Elsworth JD Lee EJK Roth RH. Divergent effects of putative anxiolytics on stress-induced fos expression in the mesoprefrontal system of the rat. Synapse. (2000) 36:143–54. 10.1002/(SICI)1098-2396(200005)36:2<143::AID-SYN7>3.0.CO;2-H 10767061

[63] Brown SM Henning S Wellman CL. Mild, short-term stress alters dendritic morphology in rat medial prefrontal cortex. Cereb Cortex. (2005) 15:1714–22. 10.1093/cercor/bhi048 15703248

[64] Gárate I García-Bueno B Madrigal JLM Caso JR Alou L Gómez-Lus ML. Toll-like 4 receptor inhibitor TAK-242 decreases neuroinflammation in rat brain frontal cortex after stress. J Neuroinflammation. (2014) 11:8. 10.1186/1742-2094-11-8 24410883 PMC3897306

[65] Zlatković J Filipović D. Chronic social isolation induces NF-κB activation and upregulation of iNOS protein expression in rat prefrontal cortex. Neurochem Int. (2013) 63:172–9. 10.1016/j.neuint.2013.06.002 23770205

[66] Amiri S Haj-Mirzaian A Rahimi-balaei M Razmi A Kordjazy N Shirzadian A. Co-occurrence of anxiety and depressive-like behaviors following adolescent social isolation in male mice; possible role of nitrergic system. Physiol Behav. (2015) 145:38–44. 10.1016/j.physbeh.2015.03.032 25817356

[67] Gilhotra N Jain H Dhingra D. Differential effects of nitric oxide synthase inhibitors on anxiety in unstressed and stressed mice. Indian J Exp Biol. (2010) 48:365–72. 20726334

[68] Moncada S Palmer RM Higgs EA. Nitric oxide: physiology, pathophysiology, and pharmacology. Pharmacol Rev. (1991) 43:109–42. 1852778

[69] Prast H Philippu A. Nitric oxide as modulator of neuronal function. Prog Neurobiol. (2001) 64:51–68. 10.1016/S0301-0082(00)00044-7 11250062

[70] Garthwaite J. NO as a multimodal transmitter in the brain: discovery and current status. Br J Pharmacol. (2019) 176:197–211. 10.1111/bph.14532 30399649 PMC6295412

[71] Chen HJC Spiers JG Sernia C Lavidis NA. Response of the nitrergic system to activation of the neuroendocrine stress axis. Front Neurosci. (2015) 9:3. 10.3389/fnins.2015.00003 25653586 PMC4300918

[72] Brown GC Bal-Price A. Inflammatory neurodegeneration mediated by nitric oxide, glutamate, and mitochondria. Mol Neurobiol. (2003) 27:325–55. 10.1385/MN:27:3:325 12845153

[73] Gambino G Rizzo V Giglia G Ferraro G Sardo P. Cannabinoids, TRPV and nitric oxide: the three ring circus of neuronal excitability. Brain Struct Funct. (2020) 225:1–15. 10.1007/s00429-019-01992-9 31792694

[74] Zareba-Koziol M Bartkowiak-Kaczmarek A Figiel I Krzystyniak A Wojtowicz T Bijata M. Stress-induced Changes in the S-palmitoylation and S-nitrosylation of Synaptic Proteins. Mol Cell Proteomics. (2019) 18:1916–38. 10.1074/mcp.RA119.001581 31311849 PMC6773552

[75] Zhang Y Deng Y Yang X Xue H Lang Y. The relationship between protein S-Nitrosylation and human diseases: a review. Neurochem Res. (2020) 45:2815–27. 10.1007/s11064-020-03136-6 32984933

[76] Nakamura T Lipton SA. Protein S-Nitrosylation as a therapeutic target for neurodegenerative diseases. Trends Pharmacol Sci. (2016) 37:73–84. 10.1016/j.tips.2015.10.002 26707925 PMC4698225

[77] Campos AC Piorino EM Ferreira FR Guimarães FS. Increased nitric oxide-mediated neurotransmission in the medial prefrontal cortex is associated with the long lasting anxiogenic-like effect of predator exposure. Behav Brain Res. (2013) 256:391–7. 10.1016/j.bbr.2013.08.006 23948217

[78] Wang D An SC Zhang X. Prevention of chronic stress-induced depression-like behavior by inducible nitric oxide inhibitor. Neurosci Lett. (2008) 433:59–64. 10.1016/j.neulet.2007.12.041 18248896

[79] Lipina C Hundal HS. The endocannabinoid system: “NO” longer anonymous in the control of nitrergic signalling? J Mol Cell Biol. (2017) 9:91–103. 10.1093/jmcb/mjx008 28130308 PMC5439392

[80] Béchade C Colasse S Diana MA Rouault M Bessis A. NOS2 expression is restricted to neurons in the healthy brain but is triggered in microglia upon inflammation. GLIA. (2014) 62:956–63. 10.1002/glia.22652 24615726

[81] Morris G Walder K Berk M Carvalho AF Marx W Bortolasci CC. Intertwined associations between oxidative and nitrosative stress and endocannabinoid system pathways: relevance for neuropsychiatric disorders. Prog Neuropsychopharmacol Biol Psychiatry. (2022) 114:110481. 10.1016/j.pnpbp.2021.110481 34826557

[82] Eljaschewitsch E Witting A Mawrin C Lee T Schmidt PM Wolf S. The endocannabinoid anandamide protects neurons during CNS inflammation by induction of MKP-1 in microglial cells. Neuron. (2006) 49:67–79. 10.1016/j.neuron.2005.11.027 16387640

[83] Lou ZY Yu WB Chen J Li L Jiang LS Xiao BG. Neuroprotective effect is driven through the upregulation of CB1 receptor in experimental autoimmune encephalomyelitis. J Mol Neurosci. (2016) 58:193–200. 10.1007/s12031-015-0656-9 26411568

[84] Kokkola T Savinainen JR Mönkkönen KS Retamal MD Laitinen JT. S-nitrosothiols modulate G protein-coupled receptor signaling in a reversible and highly receptor-specific manner. BMC Cell Biol. (2005) 6:21. 10.1186/1471-2121-6-21 15850493 PMC1090567

[85] W?dzony K Chocyk A. Cannabinoid CB1 receptors in rat medial prefrontal cortex are colocalized with calbindin- but not parvalbumin- and calretinin-positive GABA-ergic neurons. Pharmacol Rep. (2009) 61:1000–7. 10.1016/S1734-1140(09)70161-6 20081234

[86] Witkin JM Tzavara ET Nomikos GG. A role for cannabinoid CB1 receptors in mood and anxiety disorders. Behav Pharmacol. (2005) 16:315–31. 10.1097/00008877-200509000-00005 16148437

[87] Buskila Y Amitai Y. Astrocytic iNOS-dependent enhancement of synaptic release in mouse neocortex. J Neurophysiol. (2010) 103:1322–8. 10.1152/jn.00676.2009 20071630

[88] Mondelli V Vernon AC Turkheimer F Dazzan P Pariante CM. Brain microglia in psychiatric disorders. Lancet Psychiatry. (2017) 4:563–72. 10.1016/S2215-0366(17)30101-3 28454915

[89] Zhou QG Zhu LJ Chen C Wu HY Luo CX Chang L. Hippocampal neuronal nitric oxide synthase mediates the stress-related depressive behaviors of glucocorticoids by downregulating glucocorticoid receptor. J Neurosci. (2011) 31:7579–90. 10.1523/JNEUROSCI.0004-11.2011 21613472 PMC6633122

[90] Duffy SS Hayes JP Fiore NT Moalem-Taylor G. The cannabinoid system and microglia in health and disease. Neuropharmacology. (2021) 190:108555. 10.1016/j.neuropharm.2021.108555 33845074

[91] Maldonado R Cabañero D Martín-García E. The endocannabinoid system in modulating fear, anxiety, and stress. Dialogues Clin Neurosci. (2020) 22:229–39. 10.31887/DCNS.2020.22.3/rmaldonado 33162766 PMC7605023

